# Gut-Lung Microbiota Characterization in Patients with Non-Small Cell Lung Carcinoma and COVID-19 Coinfection

**DOI:** 10.34172/aim.2024.11

**Published:** 2024-02-01

**Authors:** Bahareh Vakili, Parisa Shoaei, Kiana Shahzamani, Seyed Davar Siadat, Hasan Shojaei, Zahra Esfandiari, Elahe Nasri, Shiva Shabani, Ali Zamani Moghadam, Behrooz Ataei

**Affiliations:** ^1^Infectious Diseases and Tropical Medicine Research Center, Isfahan University of Medical Sciences, Isfahan, Iran; ^2^Nosocomial Infection Research Center, Isfahan University of Medical Sciences, Isfahan, Iran; ^3^Hepatitis Research Center, School of Medicine, Lorestan University of Medical Sciences, Khoramabad, Iran; ^4^Department of Mycobacteriology and Pulmonary Research, Pasteur Institute of Iran, Tehran, Iran; ^5^Microbiology Research Center (MRC), Pasteur Institute of Iran, Tehran, Iran; ^6^Department of Microbiology, School of Medicine, Isfahan University of Medical Sciences, Isfahan, Iran; ^7^Department of Food Science and Technology, Nutrition and Food Security Research Center, School of Nutrition and Food Science, Isfahan University of Medical Sciences, Isfahan, Iran; ^8^Department of Infectious Diseases, School of Medicine, Arak University of Medical Sciences, Arak, Iran

**Keywords:** COVID-19, Gut-lung microbiota, Molecular characterization, Non-small cell lung cancer, Real-time PCR

## Abstract

**Background:**

Non-small cell lung cancer (NSCLC) patients with COVID-19 have an excessive chance of morbidity and mortality. The fecal-nasopharyngeal microbiota compositions of NSCLC patients were assessed in this study.

**Methods:**

In total, 234 samples were collected from 17 NSCLC patients infected with COVID-19, 20 NSCLC patients without confirmed COVID-19, 40 non NSCLC patients with COVID-19, and 40 healthy individuals.

**Results:**

In lung microbiota, the abundance of *Streptococcus* spp. in NSCLC patients with confirmed COVID-19 was significantly higher than the two control groups. *Pseudomonas aeruginosa* and *Staphylococcus aureus* were listed as the most frequent pulmonary bacterial groups that colonized COVID-19 patients. In fecal specimens, the numbers of Bacteroidetes, Firmicutes, and Actinobacteria phyla were significantly higher amongst NSCLC patients with COVID-19. NSCLC patients infected with COVID-19 showed lower levels of *Lactobacillus* spp., *Akkermansia muciniphila*, and *Bifidobacterium* spp. The counts of *Streptococcus* spp., in NSCLC patients with COVID-19 were significantly higher than those of healthy individuals (8.49±0.70 log CFU/g wet feces vs 8.49±0.70 log CFU/g wet feces). *Prevotella* spp. were enriched in the gut and respiratory tracts of COVID-19 patient groups. The unbiased analysis showed an increment in *Enterococcus* spp., *Streptococcus* spp., and *Prevotella* spp.

**Conclusion:**

Eventually, it was found that compared to control groups, COVID-19 patients with NSCLC showed diminished gut bacteria diversity and increase in *Lactobacillus spp*., *A. muciniphila*, and *Bifidobacterium* spp. The overgrowth of *Enterococcus* spp., *Streptococcus* spp., and *Prevotella* spp. could be potential predictive biomarkers in the gut-lung axis of NSCLC patients with COVID-19.

## Introduction

 The coronavirus infectious disease 2019 (COVID-19) resulted in a pandemic with a broad range of respiratory and gastrointestinal manifestations which contributed to more than 3.7 million global deaths.^1^ Trillions of symbiotic microbial cells inhabit human body parts, notably the skin, gastrointestinal (GI) tract and respiratory tract. Dysbiosis disrupts immune homeostasis because of an imbalance in microflora.^2^ Gut dysbiosis is associated with different GI and non-GI comorbidities, like inflammatory bowel disease, colorectal cancer, diabetes, hypertension, or obesity.^3-5^ The incidence of digestive symptoms in COVID-19 cases is reported from 2% to 50%; consequently, the GI tract is responsible for disease severity, and viral transmission.^6^

 The dominant gut microbial population of healthy adults incorporates the phyla Firmicutes, Bacteroidetes, and predominant genera include *Enterococcus, Faecalibacterium, Bacteroides, *and *Prevotella.*^1^ In the microbial communities of healthy lungs, the dominant species including Bacteroidetes, Firmicutes, Proteobacteria, and the prevailing genera incorporate *Streptococcus, Fusobacterium, Pseudomonas, Veillonella, *and* Prevotella*.^7,8^ Balanced gut microbiota affects our health notably, but dysbiosis of the gut microbial population results in pathobiont proliferation and reduced commensal bacteria that cause different local and long-lasting diseases.^1,5,7^

 Lung diseases impact the gut microbiota; in this way, a gut-lung axis permits the movement of endotoxins, cytokines, and microbial metabolites into the blood circulation and links the gut niche with that of one of the lungs.^5,9^

 In COVID-19 patients with diarrhea, the gut-lung microbiota axis involves the circulatory and immune systems.^10,11^ The immune system signals modulate bacterial patterns particularly and increment the number of *Escherichia coli or Pseudomonadales.*^12^

 COVID-19 patients with cancer are at great risk of serious morbidity and mortality.^13^ The diversity of gut microbiota is mainly reduced in elderly patients because of the leaking of SARS-CoV-2, hence COVID-19 has been more severe in this vulnerable groups by enabling SARS-CoV-2 to leak into the bloodstream and reach the internal organs.^4^ Although bacterial metabolites and fragments can modulate the lung immunity, altered gut and lung microbiota in COVID-19 patients can adjust the risk of developing severe respiratory complications, secondary bacterial infections, and death in those patients.^14-16^ However, data specifically focused on the effects of respiratory microbiota on the gut, the composition of lung microbiota in COVID-19 patients with lung cancer, and the principal risk factors for lung cancer patients with COVID-19 are scarce.^14,17^ About 1.6 million lung cancer patients succumb each year to the disease, of which non-small cell lung cancer (NSCLC) is the prevalent type of lung cancer histology (induced by acquired environmental effects and host genetic factors).^17^ Lung cancer describes a unique condition of cumulative risks for COVID-19 problems, such as advanced age, numerous comorbidities, prolonged smoking, lung tumors, and immunosuppressive treatment.^13,18^ Subsequently, lung cancer patients may be more defenseless against viral infections with poor prognosis if COVID-19 is suspected.^13^ Several studies from China, the United States, and Europe have detailed wide ranges of coinfection, from 2% to 80%.^19^ We utilized quantitative real-time polymerase chain reaction (qRT-PCR) in detecting targeted respiratory and gut bacteria; however, the use of next-generation sequencing (NGS) has low heterogeneity in the detection methods and could reveal coinfections that may be missed. We conducted a case-control study on NSCLC patients. The study included four groups of participants: 1^st^ group; NSCLC patients, 2^nd^ group; NSCLC patients infected with COVID-19 and 3^th^ Group; COVID-19 patients and 4^th^ group; healthy individuals.

 The principle objective of the current study was to investigate the gut and lung microbiota of NSCLC cancer patients and whether or not the presence of SARS-CoV-2 could alter their resident microbiota.

## Materials and Methods

###  Study Design and Base Line Characteristics

 This case-control research was performed over 12 months at the referral cancer hospital of Isfahan University of Medical Sciences, Isfahan, Iran. It is a specialized care hospital for the treatment of hematology-oncology patients. This well-equipped 166-bed center has provided specialized medical care to the community for over 50 years

 This study included 17 NSCLC patients infected with COVID-19, 20 NSCLC patients without COVID-19 infection, 40 COVID-19 patients without cancer, and 40 healthy persons who were enrolled from the beginning of April 2020 to the end of March 2021 (1:2 matched positives NSCLC to negative NSCLC). COVID-19 patients without cancer and healthy individuals were randomly selected; in addition, their sex and age were matched to NSCLC patients infected with COVID-19.

###  Inclusion and Exclusion Criteria for Patients

 All hospitalized NSCLC patients were included in the study, regardless of when cancer had been identified and only based on the persistence of SARS-CoV-2 and a certain clinical consequence (discharge or death). The survey was carried out before the start of general vaccination against COVID-19 epidemics and the participants did not receive any dose of the COVID-19 vaccines.

 NSCLC patients with evidence of surgery or neoadjuvant chemotherapy were included.NSCLC patients with acute digestive infection in the past two months who required antibiotic treatment or with a history of previous intestinal cancer treated by radiotherapy or surgery were not included. NSCLC patients with previous oropharyngeal, gastrectomy, or patients who were moved to other hospitals were also excluded.

 Healthy control individuals were chosen from people with negative results of real-time reverse transcriptase-polymerase chain reaction (RT-PCR) for SARS-CoV-2 tests (Rotor-Gene Q 6000, Qiagen, Hilden, Germany) who were referred to the reference laboratories of Isfahan. COVID-19 patients without cancer and healthy control subjects were without any disorder, pregnancy, chemotherapeutic or antimicrobial therapy for more than 3 months prior to sampling. Healthy individuals were defined as never smokers, without any respiratory diseases proved by normal chest computed tomography (CT) images or chronic disorders, within one year. CT scans were evaluated due to the presence of disorders such as ground-glass opacities in the lung. A total of 234 specimens, including 117 nasal swabs, and 117 feces samples, were gathered from the patients by professionals at the hospital. The nasopharynx and fecal samples were directly placed into sterile containers and then immediately transported to the laboratory of Infectious Diseases and Tropical Medicine Research Center, Isfahan, Iran, and stored long-term at − 80 °C. These samples were then utilized for profiling the gut and nasopharyngeal microbiota in all patients and healthy controls. The study was limited to individuals who met the specified inclusion criteria to reduce the effects of potential confounders including a food frequency questionnaire (FFQ).

###  Data Collection 

 Laboratory data (including hemoglobin, white blood cells, neutrophils, lymphocytes, platelets, albumin, creatinine, D-dimer, C-reactive protein, procalcitonin) were all collected at the time of admission (Tables S1 and S2). Data on comorbidities (diabetes, hypertension, cardiovascular disease), radiographic and pathologic findings, CT scans and lung cancer status, were collected from medical records (Tables 1 and 2).

**Table 1:** Comparison of Clinical Characteristics of NSCLC Patients with and without COVID-19

**Variables**	**NSCLC Patients with COVID-19 (n=17)**	**NSCLC Patients without COVID-19 (n=20)**	***P*** ** Value**
History of smoking	15 (88.2)	18(90)	0.63
Comorbidities			
Hypertension	5 (29.4)	7 (35)	0.49
Diabetes	1 (5.9)	2 (10)	
Cardiovascular disease	3 (17.6)	5 (25)	0.44
Others (kidney, liver)	-	2 (10)	0.28
Clinical stage^*^			
I	7 (41.2)	14 (70)	0.25
II	1 (5.9)	-	
III	3 (17.6)	3 (15)	
IV	6 (35.3)	3 (15)	
Previous hospitalization within 2 months	7 (41.2)	4 (20)	0.15
History surgery within 3 months	5 (29.4)	3 (15)	0.25
History of pneumonitis	4 (23.5)	3 (15)	0.41
Use of immunomodulatory drug within 2 months	4 (23.5)	6 (30)	0.47
Use of corticosteroids within 2 months	6 (35.3)	5 (25)	0.37
Chemotherapy treatment within 4 weeks before admission	11(64.7)	6 (30)	0.04

n/N (%) the number of patients with available data. Chi-square test for differences between two groups. ^*^Stage groups for NSCLC. Stage I: a small tumor that has not spread to any lymph nodes, Stage II: tumors (size:4-5 cm) can be removed with surgery, but often additional treatments are recommended, Stage III: have often spread extensively to the lymph nodes, but have not spread to other distant parts of the body, Stage IV: the lung cancer has spread to more than 1 area in the other lung, heart or distant parts of the body through the bloodstream.

**Table 2:** Comparison of Clinical Characteristics of NSCLC Patients and COVID-19 Patients

**Characteristics **	**NSCLC Patient with COVID-19 (n=17)**	**COVID-19 Patients (n=40)**	***P*** ** Value**
Clinical presentation			
Fever	13 (76.47)	26 (65)	0.30
Cough	14 (82.35)	19 (47.5)	0.01
Dyspnea (respiratory symptoms)	9 (52.94)	11 (27.5)	0.06
Vomiting	3 (17.64)	10 (25)	0.41
GI symptoms (Diarrhea)	3 (17.64)	5 (12.5)	0.45
Median spo_2_ %	83 (87-80)	91 (94-85)	0.04
Ground-glass opacity	14 (82.35)	22 (55)	0.04
Hospitalization			
Admission to ICU	9 (52.9%)	9 (22.5)	0.03
Oxygen therapy			
Need for supplement O_2_	10 (58.82)	18 (45)	0.25
Invasive mechanical ventilation	7 (41.17)	10 (25)	0.04
Pneumonia- Non-sever	10 (58.82)	31 (77.5)	0.13
COVID-19 severity			
Mild	1 (5.88)	8 (20)	0.19
Moderate	7 (41.17)	23 (57.5)	
Sever	9 (52.9)	9 (22.5)	
Drug administration of COVID-19			
Antibiotics	15 (88.23)	21 (52.5)	0.01
Antiviral	14 (82.35)	12 (30)	< 0.001
Median number of antibiotics administered	5 (3-6)	2 (1-3)	0.05
Median number of days exposed to treatment antibiotics (duration of antibiotic exposure)	16 (5-23)	6 (3-15)	0.04
Median duration of hospital stay	18 (5-31)	9 (4-21)	0.04

n/N (%) the number of patients with available data. Chi- square test for differences between two groups.

###  Definitions

 Diagnosis of COVID-19 was based on WHO’s temporary guideline.^20^ COVID-19 pneumonia was confirmed by clinical criteria and laboratory nucleic acid detection confirmed on genes targeted N and ORF1ab.^21^

 COVID-19 patients were categorized in three groups, moderate: patients with respiratory symptoms of pneumonia; severe: patients with shortness of breath, and venous oxygen saturation < 93% in resting state; and critical: hospitalized patients with acute respiratory distress.

 Colonization was defined as the presence of microorganisms, including detection of the bacteria (threshold of 10^2^ CFU/mL). Above this threshold, some microbial flora discerns potential pathogens in immunocompetent.^22^

 The quantities of respiratory pathogens including *Staphylococcus aureus*, *Streptococcus pneumoniae*, *Haemophilus influenza*,* Pseudomonas aeruginosa,* and *Moraxella catarrhalis *in COVID-19 patients groups were taken into account. Moreover, other bacterial agents belonging to the gastrointestinal or nasopharyngeal flora such asBacteroidetes, Firmicutes, Actinobacteria phyla, *Prevotella* spp., *Veillonella* spp., *Streptococcus* spp.,* Enterococcus *spp., *Enterobacteriaceae, Lactobacillus *spp*., Clostridium *spp.*, Bifidobacterium *spp.,* Fusobacterium *spp.*, Faecalibacterium prausnitzii, *and *Akkermansia muciniphila* were considered. Nasopharyngeal swabs and stool samples were examined for the qRT-PCR based on different targeted bacteria quantities as described previously.^23^

###  Total DNA Isolation 

 Total DNA extraction from 500 microliters of nasopharyngeal swabs and 220 mg of stool was performed using QIAmp DNA stool and Blood mini kits (Qiagen) following the manufacturer’s instructions.^24^ DNAs were purified and stored at –80 °C for further experiments. DNA concentration was quantified using the Qubit^TM^ 4Florometer (Life Tech, Invitrogen, Singapore) before performing qRT-PCR.

###  Quantitative Real-Time PCR Analysis

 The SYBR Green PCR Master Mix (Applied Biosystems) in Rotor-Gene 6000 real-time PCR cycler (Qiagen Corbett, Hilden, Germany), using the unique QuantiTect SYBR® Green PCR kit qPCR master mix (Yekta Tajhiz Azma Co, Tehran, Iran) and the proper qPCR primers, determined the bacte­rial load based on the abundance of 16S rRNA gene (Supplementary file 1, Table S3).^24-29^

 About 50 ng of DNA from fecal and nasopharyngeal swabs (containing targeted species DNA), 1 × SYBR green qPCR master mix, and 0.5 μM of each primer were applied in each qPCR assay to generate standard curves for the enumeration of target DNA in test samples. All tests were assessed in duplicate, and the mean values were evaluated, as well. Every run contained non-template and positive controls. Amplification was performed under the following conditions: 95 °C for 5 minutes, followed by 40 cycles of denaturation at 95 °C for 1 minute, 30 s at the appropriate annealing temperature and 72 °C for 1 minute, and the final step extension at 72 °C for 5 minutes. The melting curve analysis was performed after qPCR with constant fluorescence inspection under a gradual heating rate of 0.1 °C/s from 72 to 95 °C. Standard curves were generated for assessment of the number of bacterial strains in samples with serially diluted solutions of the total extracted DNA from the reference strains. This method is reliable and consistent, and its accuracy is appropriate.

 The bacterial concentrations (from fecal and nasopharyngeal swabs) were calculated by competitive cycle threshold values and presented as DNA copy numbers in reactions. Table S4 supplies standard curve parameters such as strains of bacteria, qPCR amplification efficiency, and determination coefficient. The correlation coefficient values of the standard curve ranged from 0.99 to 1.0.

###  Statistical Analysis 

 The SPSS software version 21.0 (SPSS Inc. Chicago, IL, USA) was performed for statistical calculations. Values were estimated as mean ± standard deviation (SD). The Shapiro–Wilk test was applied for testing the normality. The Q-Q plot was also performed for assumption of the normality. One-way analysis of variance (ANOVA) and Pearson’s chi-square were used for continuous variables (e.g. body mass index, age, and inter-patient comparisons) and categorical variables, respectively. A *P *value < 0.05 was considered statistically significant. We performed a post hoc test to identify exactly the differences between groups. ANOVA (Bonferroni’s, Dunnett’s tests) was performed in multiple comparisons between the groups. Box-plot graphs showed the median values of qPCR results as lines across the box. A t-test was performed to compare the means of the two groups of COVID-19 patients without cancer and healthy control subjects.

## Results

**Figure 1:**
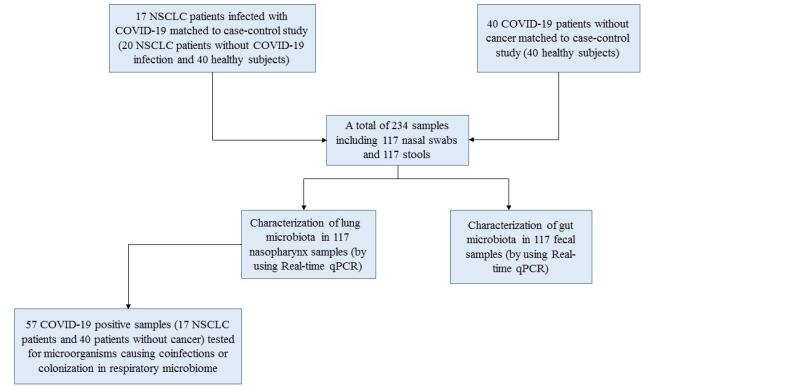
Flow Diagram Summarizing the Information of Samples and Patients in This Study

 A total of 234 samples were evaluated including 117 nasal swabs, and 117 feces samples from the patients and the healthy control group (Figure 1). The clinical characteristics and basic information of studied patients are shown in Tables 1 and 2. The mean age of NSCLC patients with COVID-19 was 58.7 (range 53.3-64.1), and 82.3% of them were male. They represented considerably higher coughs and received more antibiotics and antiviral agents compared to NSCLC patients without COVID-19 (*P* < 0.01, *P* < 0.01, and *P* 0.001, respectively).

 No significant difference was found in terms of BMI, age, or COVID-19 severity between patient groups and the control group (Table S5). Results revealed that the rate of chemotherapy treatment 4 weeks before admission in COVID-19 patients with NSCLC was significantly more than the NSCLC patients with negative COVID-19 (*P* = 0.04). CT images in NSCLC patients with COVID-19 showed more ground-glass opacity than patients with COVID-19 (*P* = 0.04). FFQ analysis showed no significant differences between the studied groups when they were compared for dietary intake of proteins and carbohydrates and fat.

###  Quantitation of Lung Microbiota 

**Figure 2:**
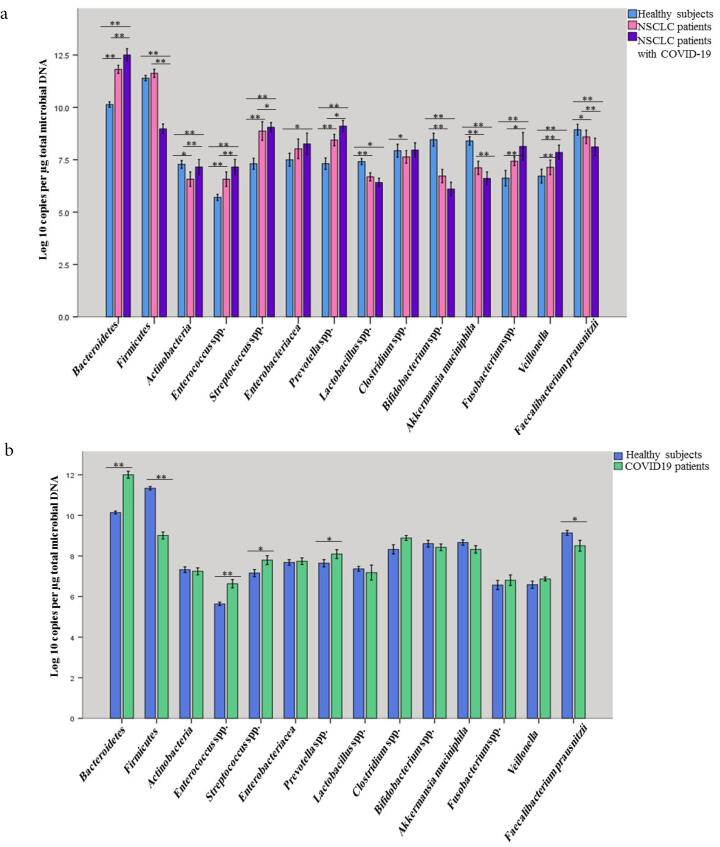
(a) Comparing the presence of targeted members of gut microbiota of studied groups, including NSCLC (non-small cell lung cancer) patients, NSCLC patients with COVID-19 and healthy subjects. (b) Comparing the presence of targeted members of gut microbiota of studied groups, including COVID-19 patients and healthy subjects. Data of qPCR are presented as mean log10 copies/µg of total microbial DNA. In these diagrams, the error bar indicates mean ± SD, 95% CI, **P < 0.01, *P < 0.05

 A comparison of quantities of selected microbial groups in lung microbiota in studied patients and healthy people is shown in Figure 2 and Table 3 (Table S6). Table 3 shows the average counts and the standard deviation of lung-targeted microorganisms. It showed that the plenitude of *Streptococcus *spp. in NSCLC patients suffering from COVID-19 was significantly higher than healthy subjects and NSCLC patients (157 ± 49.01; *P* < 0.003, 126 ± 34.71; *P* < 0.001, and141 ± 47.56; *P* < 0.002, respectively) (Table 3 and Figure 2). An expanded quantity of *Prevotella* spp. was found in COVID-19 patients with and without NSCLC (*P* < 0.05). *P. aeruginosa *and* S. aureus *were listed as the most frequent pulmonary bacterial groups colonizing in COVID-19 patients (Table 4). Furthermore, *P. aeruginosa *colonization was revealed in the lung microbiota of 35.3% (6/17) of NSCLC patients suffering from COVID-19.

**Table 3:** Comparison of Population Numbers of Selected Microbial Groups in the Lung of Healthy Control, NSCLC Patients, and NSCLC Patients with COVID-19

**Target Bacterial **	**Healthy Control** **(n=40)**	**NSCLC Patients ** **(n=20)**	**NSCLC Patients with COVID-19 (n=17)**	***P*** ** Value**		
				**A**	**B**	**C**
Bacteroidetes	3140 ± 768.54	2235 ± 534.3	2470.5 ± 908.8	< 0.001	0.009	< 0.001
Firmicutes	4540.5 ± 1001.74	3655 ± 820.4	3900 ± 861.68	0.003	0.060	< 0.001
Actinobacteria	130 ± 37.96	112 ± 33.49	141.93 ± 29.74	0.197	0.295	< 0.001
*Prevotella* spp.	2.12 ± 0.143	2.16 ± 0.166	2.26 ± 0.232	< 0.001	0. 261	0.026
*Veillonella*	114.2 ± 43.37	154 ± 36.03	133.11 ± 41.5	0.061	0.382	< 0.001
*Streptococcus *spp.	126 ± 34.71	141 ± 47.56	157 ± 49.01	0.003	< 0.001	0.002

A, NSCLC patients vs. Healthy control; B, NSCLC patients with COVID-19 vs. Healthy control; C, NSCLC patients with COVID-19 vs. NSCLC patients. Relationships among between the groups were performed assessed using the ANOVA test in multiple comparisons. Values were presented as mean ± standard deviation (SD). *P* values < 0.05 was considered statistically significant.

**Table 4:** Copies/mL of Colonized Bacteria in Studied Patients (NSCLC Patients with COVID-19 and COVID-19 Patients Without NSCLC)

**Bacterial **	**Patient type**	**≥10**^2^ ** copies/mL** **No. (%)**	**≥10**^2^ ** -<10**^5^ ** copies/mL** **No. (%)**	**≥10**^5^ ** copies/mL** **No. (%)**
*S. aureus*	NSCLC patients with COVID-19 (n = 17)	1	-	-
	Patients with COVID-19 (n = 40)	-	1	1
*S. pneumonia*	NSCLC patients with COVID-19 (n = 17)	-	-	-
	Patients with COVID-19 (n = 40)	-	1	-
*M. catarrhalis*	NSCLC patients with COVID-19 (n = 17)	-	1	-
	Patients with COVID-19 (n = 40)	-	-	-
*H. influenza*	NSCLC patients with COVID-19 (N = 17)	-	-	1
	Patients with COVID-19 (N = 40)	-	-	-
*P. aeruginosa*	NSCLC patients with COVID-19 (N = 17)	2	-	2
	Patients with COVID-19 (n = 40)	1	2	-

Data are presented as Log copies/mL. Absent (Log copies /mL < 10^2^), Present (Log CFU/mL 10^2^-10^5^).

###  Quantitation of Fecal Microbiota by qPCR 

**Figure 3:**
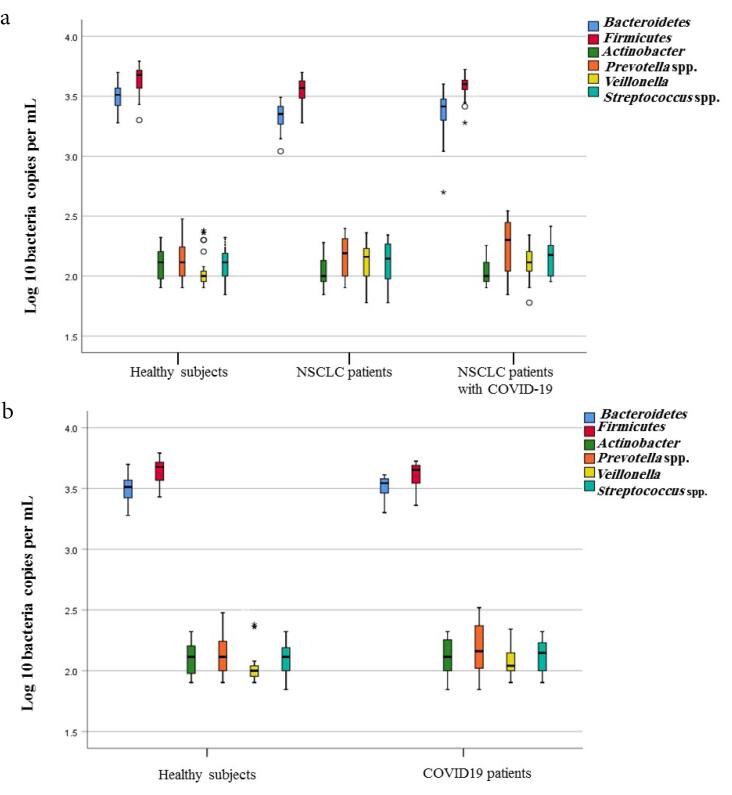
Box-and-Whisker Plots of Bacterial Groups Quantified by qPCR. Bacterial groups quantified by SYBR Green qPCR and expressed as Log10 bacteria per mL nasopharyngeal in the lung microbiota of studied groups. **(a)** Comparing the presence of targeted members of lung microbiota of studied groups, including NSCLC (non-small cell lung cancer) patients, NSCLC patients with COVID-19 and healthy subjects. **(b)** Comparing the presence of targeted members of lung microbiota of studied groups, including COVID-19 patients and healthy subjects

 Quantities of Bacteroidetes, Firmicutes, Actinobacteriaphyla,* Enterococcus *spp.*, Streptococcus *spp.*, Enterobacteriaceae, Prevotella* spp.,* Lactobacillus *spp.,* Clostridium *spp.,* Bifidobacterium *spp., *F. prausnitzii, Fusobacterium *spp.,* A. muciniphila,* and*Veillonella* were detected in all fecal specimens; however, the numbers of Bacteroidetes, Firmicutes, and Actinobacteriaphyla were significantly (*P* < 0.001, Table 5, Figure 3) higher among NSCLC patients with COVID-19 compared with COVID-19 patients and healthy subjects. NSCLC patients with COVID-19 had lower levels of *Lactobacillus *spp., *A. muciniphila*, and *Bifidobacterium* spp. compared to COVID-19 patients and healthy control group (*P* < 0.001). Our results confirmed that COVID-19 patients showed a significant decline in Firmicutes (*P* = 0.00), *Prevotella* spp., (*P* = 0.015), *F. prausnitzii* (*P* = 0.013) while increased quantities of Bacteroidetes, (*P* < 0.001), *and Enterococcus *spp., (*P* < 0.001), *Streptococcus *spp., (*P* = 0.021) were observed in comparison with healthy subjects (Figure 3 and Table S7). The counts of *Streptococcus* spp., in the lung and gut, had been notably (*P* < 0.05) higher in the NSCLC patients suffering from COVID-19 (8.49 ± 0.70 log CFU/g wet feces) than healthy controls (7.15 ± 0.55 log CFU/g wet feces) (Table 5, Supplementary Tables S6 and S7). Of note, *Prevotella* spp. was also enriched in the gut and respiratory tracts of COVID-19 patients with or without lung cancer (*P* < 0.05). The differences in intestinal bacterial of COVID-19 patients are illustrated in Figures S1 and S2.

**Table 5:** Comparison of Population Numbers of Selected Microbial Groups in Healthy Control, NSCLC Patients, and NSCLC Patients with COVID-19

**Target bacterial **	**Healthy Control** **(n=40)**	**NSCLC Patients ** ** (n=20)**	**NSCLC Patients ** **with COVID-19 ** **(n=17)**	***P*** ** Value**		
				**A**	**B**	**C**
Bacteroidetes	10.13 ± 0.23	11.7 ± 0.37	12.5 ± 0.59	< 0.001	< 0.001	< 0.001
Firmicutes	11.34 ± 0.46	10.89 ± 0.19	8.97 ± 0.45	0.068	0 < 0.001	< 0.001
Actinobacteria	7.32 ± 0.34	7.05 ± 0.28	5.68 ± 0.25	0.034	< 0.001	< 0.001
*Enterococcus *spp.	5.63 ± 0.27	6.53 ± 0.64	7.15 ± 0.70	< 0.001	< 0.001	< 0.001
*Streptococcus *spp.	7.15 ± 0.55	7.9 ± 1.00	8.49 ± 0.70	< 0.001	< 0.001	0.015
*Enterobacteriaceae*	7.68 ± 0.43	7.92 ± 0.88	8.25 ± 1.00	0.658	0.023	0.522
*Prevotella* spp.	7.64 ± 0.61	8.47 ± 0.15	9.10 ± 0.53	< 0.001	< 0.001	0.021
*Lactobacillus *spp.^*^	3.04E7 ± 1.91E7	6.49E6 ± 5.27E6	3.78E6 ± 9.81E5	0.002	0.051	0.981
*Clostridium *spp.	8.23 ± 0.71	7.52 ± 0.16	7.96 ± 0.67	0.05	0.207	0.158
*Bifidobacterium *spp.	8.60 ± 0.53	6.5 ± 0.74	6.09 ± 0.63	< 0.001	0.003	0.112
*F. prausnitzii*	9.13 ± 0.40	8.57 ± 0.29	8.11 ± 0.80	0.05	< 0.001	0.003
*Fusobacterium *spp.	6.56 ± 0.71	7.44 ± 0.43	8.13 ± 1.29	< 0.001	< 0.001	0.045
*A. muciniphila*	8.66 ± 0.41	7.16 ± 0.58	6.60 ± 0.61	< 0.001	< 0.001	0.004
*Veillonella*	6.58 ± 0.55	7.15 ± 0.63	7.84 ± 0.86	0.003	< 0.001	0.002

A, NSCLC patients vs. Healthy control; B, NSCLC patients without COVID-19 vs. Healthy control; C, NSCLC patients with COVID-19 vs. NSCLC patients ^*^Bacteria with normal distribution. Relationships between the groups were assessed using the ANOVA test in multiple comparisons. Values were presented as mean ± standard deviation (SD). *P* values < 0.05 was considered statistically significant.

## Discussion

 Characterization of the gut and lung microbiota in patients with NSCLC, as a common cancer, particularly when complicated by SARS-CoV-2 infection, is less known.^3,13^ However, learning about the systemic influence of the gut microbiota and its relation to the immune system made a huge leap in clarifying the influence of gut microbiota on chemotherapy and lung cancer treatment.^3^

 We found that the rate of chemotherapy treatment before infection withSARS-CoV-2 in NSCLC patients was significantly higher than lung cancer patients. CT images in NSCLC patients with COVID-19 showed more ground-glass opacity than COVID-19 patients.

 Our data in accordance with other studies revealed that in lung cancer patients, cumulative risk factors including chemotherapy and ICU admission correlated with severe outcomes in COVID-19 patients.^13,14,17^

 In a healthy individual, the lung microbiota has a low population density but an important variety of interacting microbiota. Usually, there are species of the Firmicutes such as *Streptococcus* sp. and *Veillonella* sp., Bacteroidetes including *Prevotella* sp., and Proteobacteria phyla in the lung microbiota of healthy adult individuals.^2,30^ The lung microbiota is altered in NSCLC and the microbiota affects cancer development and progression.^14^

 The abundance of *Streptococcus *spp., was higher in NSCLC patients with COVID-19; additionally, an increased quantity of *Prevotella* spp. was found in COVID-19 patients.*Prevotella* and *Veillonella* were most strongly associated with NSCLC, and Veillonella significantly intensified the progression of NSCLC in lung microbiota.^17^ Persistence of *Veillonella* sp. and*Prevotella* spp. in the airways, rise their metabolism of mucus and more colonization by bacterial pathogens causing pneumonia.^31^

 Dysbiosis in the lung microbiota population such as a diminishment in Betaproteobacteria and an increment in *Staphylococcus, Streptococcus, *and *Enterobacteriaceae* correlates with the host antiviral immune response together with serum cytokine IL-6.^12,14^

 Consequently, the lung microbiota predicts the severity of disease and clinical outcomes in these patients.^14^

 The landscape of lung microbiota in COVID-19 patients showed that some pathogens, such as *Klebsiella oxytoca* causingpneumonia, colitis, and sepsis, are increased in COVID-19 patients and reduced in healthy individuals.^12,32^ According to the results, *P. aeruginosa *and* S. aureus *were the mostcommon pulmonary bacterial groups that colonized in the lung microbiota of COVID-19 patients.

 Other studies using a high-throughput sequencing procedure in analyzing the oropharyngeal microbiota of patients with pneumonia have found a significant rise in *Pseudomonas *and *Bacillus *species after influenza viral infection; however, the number of *Veillonella*, *Prevotella, *and *Neisseria *were significantly decreased.^33^ In nasopharyngeal microbiota of COVID-19 patients, taxa such as *Staphylococcus, *that were detected in our study, were shown to increase in the nasopharynx microbiota of H3N2 influenza patients in comparison to control groups.^34^

 As already detailed, there are differences between the population density and composition of the lung microbiota and that of the intestinal tract due to bidirectional movement of air and mucus^14^ Other independent studies showed that compared to healthy individuals, the diversity of the intestinal microbiota was significantly decreased in SARS COV-2 positive patients.^35^ Key taxa such as *F. prausnitzii, Bifidobacteria sp.*, Actinobacteria, *Ruminococcus sp*., and *Lachnospiraceae* were depleted but the abundance of *A. muciniphila*, *Bacteroides *sp.,*Veillonella*,* Clostridium *sp., *Enterococcus *and *Streptococcus *was increased.^1,36^ The stools of these NSCLC patients were revealed to be enriched in phylum Firmicutes, Akkermansia, and Ruminococcus (*F. prausnitzii*) genera.^3^

 Similar to another previous study, a reduction in bacterial frequency was revealed in stool samples of COVID-19 patients compared with the healthy control group. Our evidence of the intestinal microbiota indicated a significant decline in Firmicutes, *Prevotella spp.,* and *F. prausnitzii* in COVID-19 patients compared to the control groups. The intestinal bacteria could transfer to other organs through the intestinal barrier, and the COVID-19 infection could promote the switch of intestinal bacteria.^32^

*Faecalibacterium prausnitzii* produces short chain fatty acids (SCFAs) which play an important role in neuro-immunoendocrine regulation and are key sources for intestinal epithelial cells to improve the gut barrier function^37^ Besides, SCFAs activate signaling cascade by cell surface G-protein coupled receptors that control immune functions and inflammatory side effects.^1^ *F. prausnitzii*, which produces butyrate and IL-10 in the gut leads to a negative relation with COVID-19 severity and protects the host from the infection.^12,32^

 Consistent with the gut-lung axis concept, a cross-sectional study showed that the bacterial diversity of gut microbiota is significantly decreased in COVID-19 patients (with and without NSCLC). In our study, increased quantities of *Streptococcus spp.,* and *Prevotella spp.* (*P <*0.05) were found in the gut-lung axis of NSCLC patients with COVID-19 (with and without NSCLC).

*Prevotella* genusis commensal and scarcely ever involved in acute infections. However, some strains are opportunistic pathogens in chronic diseases, mucosal inflammation, and SARS-CoV-2 with activation of immune signaling pathways.^31^ The bacterial population also suggests the robustness of the lung bacterial community in limiting SARS-CoV-2 increase or attachment.

 An increased levels of bacteria belonging toFirmicutes, Actinobacteria, and Bacteroidetes phyla (*P* < 0.001), which produce SCFAs, particularly butyric acid.^12^ A lower plentitude of *Lactobacillus *spp., *A. muciniphila*, and *Bifidobacterium* spp.,was observed in NSCLC patients with COVID-19 when compared with control subjects. As stated previously, dysbiosis of the gut- lung axis including* Bifidobacterium *and* Lactobacillus* communities can increase the abundances of opportunistic pathogensin COVID-19 and systemic inflammatory response syndrome patients.^37,38^ Furthermore, patients with NSCLC or renal cell carcinoma responding to immunotherapy showed enhancement of *A. muciniphila* at diagnosis.^39^

 Woodall et al reported increased amounts of *Enterococcus* in the gut microbiota of patients with COVID-19 which is similar to this survey.^1^ Many strains of* Enterococcus *species are pathobionts and have been shown in SARS-CoV-2 infection, gastric and colorectal cancers.^35^

 A recent cohort study in COVID-19 patients revealed persistent microbial dysbiosis with previous viral clearance, regardless of antibiotics and anti-viral medications.^40^ Gut-colonizing bacteria such as *Enterobacteriales* in the respiratory tract have detrimental effects on the outcomes of respiratory diseases but increased gut-colonizing organisms were not discovered in this study.^8^ NSCLC patients who responded to neoadjuvant and chemotherapy agents harbored a higher diversity of gut microbiota at baseline with constant composition during therapy.^41^

 Some limitations of the study are as follows: Obtaining NSCLC specimens from different groups of studied patients was time-consuming; therefore, the sample size of the study should be increased in future researches. Because of limited resources of NGS technologies in developing countries, we performed the qRT-PCR based on different targeted bacteria quantities. The gut microbiota plays a crucial function in host homeostasis and promises a new anti-lung cancer approach, but there is a need to further evaluate lung microbiota and its products, both in health and in disease.

 The studied patients showed evidence of nasopharyngeal microbiota disorders connected with disease severity, and there was a significant relation between severity of COVID-19 and microbiota signatures early in the disease. There are significant relations between the duration of hospitalization, the type of oxygen support, and antibiotic treatment, which affect the microbiota of the upper respiratory tract. One of the weaknesses of the current study is that we could not measure confounding variables and adjust their effect in the model; therefore, subsequent studies should control the influence of these confounders.

 Because of the ambiguous extent of the contribution of the human microbiota to COVID-19 and lung cancer, further microbiota studies from different countries, severe clinical conditions, and longitudinal information (e.g. post-hospitalization) are required.

## Conclusion

 Finally, we found that compared to control groups, COVID-19 patients with NSCLC showed a diminishment in gut bacteria diversity and increase in *Lactobacillus *spp., *A. muciniphila*, and *Bifidobacterium* spp. Unbiased analysis indicated an increment in *Enterococcus* spp., *Streptococcus *spp., and *Prevotella* spp. in COVID-19 patients with NSCLC. Distinctive microbiota strategies and confounder effects lead to high heterogeneity across studies,which showed lower plenitude in COVID-19 patients with NSCLC than control subjects.

## Supplementary Files

Supplementary file 1 contains Tables S1-S7 and Figures S1 and S2.
