# Supplementary material for: Gut-Lung Microbiota Characterization in Patients with Non-Small Cell Lung Carcinoma and COVID-19 Coinfection
Source: Arch Iran Med. 2024 Feb 1;27(2):62–71. doi: 10.34172/aim.2024.11 (PMC11017262; doi:10.34172/aim.2024.11)

Arch Iran Med. February 2024;27(2):62-S1.

10.34172/aim.2024.11

**Supplementary file**

**Table S1**. Laboratory Data of NSCLC Patients with COVID-19 on Admission.

| **Laboratory Data** | **Reference interval** | **NSCLC patients with COVID-19 (n=17)** |
|---|---|---|
| White blood cells (WBC) | 4-11 ($\times 10^3$ /mm$^3$) | 5.3 (4.1-8.1) |
| Neutrophil | 2-7 ($10^3$ / mm$^3$) | 3.8 (2.7-6.9) |
| Lymphocyte | 0.8 - 4 ($10^3$ / mm$^3$) | 0.8 (0.3-1.1) |
| Monocyte | 0.12 - 1.2 ($10^3$ / mm$^3$) | 0.41 (0.2-0.6) |
| Platelet | 150 - 450 ($10^3$ / mm$^3$) | 191 (126-238) |
| Hemoglobin | 14-18 (g/dL) | 12.2 (9.4-13.1) |
| D-dimer | Up to 350 (ng/mL) | 500 (360-1880) |
| Creatinine | 0.7-1.4 (mg/dL) | 1.51 (0.7-1.9) |
| Alanine aminotransferase | 10-40 (U/liter) | 32 (21-38) |
| Aspartate aminotransferase | 10-40 (U/liter) | 35 (24-43) |
| C-reactive protein (CRP) | 1-6 (mg/dL) | 38.1 (4.6–103.9) |
| Procalcitonin | 0-0.5 (µg/L) | 0.13 (0.03–0.45) |

**Table S2**. Laboratory Data of Patients with COVID-19 on Admission.

| **Laboratory Data** | **Reference range** | **patients with COVID-19 (n=40)** |
|---|---|---|
| White blood cells ($\times 10^9$ cell/ml) | 4-11 ($\times 10^3$ /mm$^3$) | 4.9 (3.5-7.8) |
| Neutrophil ($\times 10^9$ cell/ml) | 2-7 ($10^3$ / mm$^3$) | 3.26 (2.39–4.67) |
| Lymphocyte ($\times 10^9$ cell/ml) | 0.8 - 4 ($10^3$ / mm$^3$) | 0.99 (0.3.7–1.31) |
| Monocyte ($\times 10^9$ cell/ml) | 0.12 - 1.2 ($10^3$ / mm$^3$) | 0.45 (0.4-0.6) |
| Platelet ($\times 10^9$ cell/ml) | 150 - 450 ($10^3$ / mm$^3$) | 201 (137-248) |
| Hemoglobin (mg/l) | 14-18 (g/dL) | 13.8 (11.3-15.3) |
| D-dimer (mg/l) | Up to 350 (ng/mL) | 4 (1.8-16.7) |
| Creatinine (mg/dl) | 0.7-1.4 (mg/dl) | 1.01 (0.6-1.1) |
| Alanine aminotransferase (U/liter) | 10-40 (U/liter) | 31 (21-36) |
| Aspartate aminotransferase (U/liter) | 10-40 (U/liter) | 33 (23-39) |

| | | |
|---|---|---|
| C-reactive protein (CRP)(mg/dL) | 1-6 (mg/dL) | 23.2 (2.4–54.9) |
| Procalcitonin | 0-0.5 (μg/L) | 0.06 (0.03–0.24) |

**Table S3.** Primers Used in this Study for SYBR Green qPCR Reactions.

| Species | Primer sequence5`-3` | Annealing temperature(°C) | Amplified fragment(bp) | References |
|---|---|---|---|---|
| *Bacteroidetes* | F- GTTTAATTCGATGATACGCGAG<br>R- TTAASCCGACACCTCACGG | 60 | 122 | 25 |
| *Firmicutes* | F - TGAAACTYAAGGAATTGACG<br>R - ACCATGCACCTGTC | 55 | 126 | 25 |
| *Actinobacteria* | F- TGTAGCGGTGGAATGCGC<br>R- AATTAAGCCACATGCTCCGCT | 60 | 277 | 26 |
| *Enterococcus* spp. | F-CCC TTA ATT GTT AGT TGC CAT CAT T<br>R-ACT CGT TGT ACT TCC CAT TGT | 56 | 144 | 27 |
| *Streptococcus* spp. | F-CTWACCAGAAAGGGACGGCT<br>R- AAGGRYCYAACACCTAGC | 60 | 170 | 28 |
| *Enterobacteriacea* | F- GTGCCAGCMGCCGCGGTAA<br>R- GCCTCAAGGGCACAACCTCCAAG | 67 | 124 | 29 |
| *Prevotella* spp. | F-CAG CAG CCG CGG TAATA<br>R-GGC ATC CAT CGT TTA CCG T | 54 | 309 | 27 |
| *Lactobacillus* spp. | F-AGC AGT AGG GAA TCT TCC A<br>R-CAC CGC TAC ACA TGGAG | 50 | 341 | 30 |
| *Clostridium* spp. | F-CGG TAC CTG ACT AAG AAG C<br>R-AGT TTY ATT CTT GCG AAC G | 50 | 429 | 30 |
| *Bifidobacterium* spp. | F-GGG TGG TAA TGC CGG ATG<br>R-TAA GCG ATG GAC TTT CAC ACC | 55 | 442 | 30 |
| *F. prausnitzii* | F-GAT GGC CTC GCG TCC GAT TAG<br>R-CCG AAG ACC TTC TTC CTC C | 58 | 198 | 31 |
| *Fusobacterium* spp. | F-C(A/T)AACGCGATAAGTAATC<br>R- TGGTAACATACGA(A/T)AGGG | 54 | 273 | 32 |
| *A. muciniphila* | F-CAG CAC GTG AAG GTG GGG AC<br>R-CCT TGC GGT TGG CTT CAG AT | 65 | 329 | 31 |
| *Veillonella* | F-A(C/T)CAACCTGCCCTTCAGA<br>R-CGTCCCGATTAACAGAGCTT | 62 | 343 | 32 |
| *Staphylococcus aureus* | F- GAGGTATTAGTGATATCGATTTAA<br>R- GCTTTAATGTCACTTGTATTGAT | 75 | 139 | 33 |
| *Streptococcus pneumoniae* | F- ACGCAACTG ACGAGTGTGAC<br>R- GATCGCGACACCGAACTAAT | 65 | 264 | 24 |
| *Pseudomonas aeruginosa* | F- CCAGCCATGCCGCGTGTGTGA<br>R- GTTGGTAACGTCAAAACAGCAAGG | 55 | 102 | 24 |

| | | | | |
|---|---|---|---|---|
| *Moraxella catarrhalis* | F-CTGCCCAAGCTGCCCTAAGT<br>R-CAAAAGCCACAAAAACCC | 57 | 134 | 34 |
| *Haemophilus influenza* | F- TTGGTGGTCAGCGTTTCGGTGAGA<br>R- CAAGTGAGCGGATTTCTTTCATAAT | 65 | 112 | 35 |

**Table S4.** Bacterial strains used for formation of standard curves, efficiency, correlation coefficients and melting temperatures of quantitative PCR.

| Target bacterial | Bacterial standard strain | Efficiency (%) | Correlation coefficient ($R^2$) | Melting temperature |
|---|---|---|---|---|
| Bacteroidetes | *B. fragilis* ATCC[*] 25285 | 98.7 | 0.999 | 87.5 |
| Firmicutes | *C. difficile* ATCC 9689 | 102.4 | 0.998 | 84.5 |
| Actinobacteria | *Mycobacterium avium* ATCC 15769 | 100 | 0.997 | 87.4 |
| *Enterococcus* spp. | *E. faecalis* ATCC 19433 | 89.9 | 0.999 | 89.5 |
| *Streptococcus* spp. | *S. pneumoniae* ATCC 49619 | 98 | 0.998 | 88.6 |
| *Enterobacteriacea* | *E.coli* ATCC 25922 | 88.4 | 0.999 | 89 |
| *Prevotella* spp. | *P. intermedia* ATCC 25611D-5 | 90.5 | 0.999 | 87.5 |
| *Lactobacillus* spp. | *L. acidophilus* ATCC 4356 | 100.5 | 0.999 | 87 |
| *Clostridium* spp. | *C. perfringens* ATCC 13124 | 99.5 | 0.999 | 88 |
| *Bifidobacterium* spp. | *B. bifidum* ATCC 29521 | 101 | 0.999 | 90.8 |
| *F. prausnitzii* | *F. prausnitzii* ATCC 27766 | 99.8 | 0.997 | 89.5 |
| *Fusobacterium* spp. | *F.* nucleatum *ATCC* 25586 | 99 | 0.998 | 89 |
| *A. muciniphila* | *A. muciniphila* ATCC BAA-835 | 104.6 | 0.997 | 90 |
| *Veillonella* | *V. parvula* ATCC 17745 | 106.2 | 0.998 | 88.8 |

[*]American Type Culture Collection.

| **Table S5**. Demographic Data of Samples Used in this Study. | | | | |
|---|---|---|---|---|
| Characteristics | NSCLC patients with COVID-19 (n=17) | NSCLC patients without COVID-19 (n=20) | Healthy control | Patients with COVID-19 |
| Subjects | 17 | 20 | 40 | 40 |
| Sex ratio (male/female) | 14/3 | 19/1 | 25/15 | 29/11 |
| Age, y, mean(SD)[a] | 58.7±5.37 | 56.3±2.48 | 52.3±16.1 | 55.9±8.14 |
| BMI(Kg/m$^2$), mean(SD)[a] | 24.52±4.3 | 26.31±21 | 26.61±4.3 | 27.13±1.18 |

[a]No significant difference between groups.

| **Table S6**. Comparison of Population Numbers of Selected Microbial Groups in COVID-19 Patients and Healthy Individuals. | | | | | | |
|---|---|---|---|---|---|---|
| **Bacterial species** | **Copies/gr of faecal** | | **Levene`s Test Equal variances assumed** | | **t-test for equality of means** | |
| | **Healthy (N=40)** | **COVID19 (N=40)** | **F** | **Sig.** | **T** | **Sig.(2-tailed)** |
| Bacteroidetes | 3140±768.54 | 3317.5±591.34 | 2.71 | 0.104 | -1.158 | 0.251 |
| Firmicutes | 4540.5±1001.74 | 4192.5±846.22 | 0.875 | 0.357 | 1.676 | 0.098 |
| Actinobacteria | 130±37.96 | 135±44.02 | 2.089 | 0.152 | -0.571 | 0.570 |
| *Prevotella* spp. | 141.7±49.60 | 171.7±76.89 | 11.96 | 0.001 | -2.074 | 0.041 |
| *Veillonella* | 114.2±43.37 | 125.2±36.01 | 0.002 | 0.965 | -1.234 | 0.221 |
| *Streptococcus* spp. | 126±34.71 | 138±39.25 | 1.169 | 0.283 | -1.507 | 0.136 |

**Table S7**. Comparison of Population Numbers of Selected Microbial Ggroups in COVID-19 Patients and Healthy Individuals.

| Target bacterial | Copies/gr of faecal | | Levene`s Test Equal variances assumed | | t-test for equality of means | |
|---|---|---|---|---|---|---|
| | **Healthy(N=40)** | **COVID19(N=40)** | **F** | **Sig.** | **T** | **Sig.(2-tailed)** |
| **Bacterial Phyla** | | | | | | |
| Bacteroidetes | 1.6E10±1.01E10 | 1.9E12±2.3E12 | 58.6 | 0.000 | -5.17 | 0.000 |
| Firmicutes | 2.66E11±4.10E11 | 3.25E9±2.89E10 | 22.3 | 0.000 | 7.94 | 0.000 |
| Actinobacteria | 6.38E8±2.24E8 | 3.74E8±4.21E9 | 3.21 | 0.077 | -0.872 | 0.389 |
| **Bacterial species** | | | | | | |
| *Enterococcus* spp. | 5.11E5±3.2E6 | 9.95E6±1.80E7 | 11.89 | 0.001 | -3.31 | 0.001 |
| *Streptococcus* spp. | 5.41E7±1.77E8 | 1.53E8±2.02E8 | 5.50 | 0.001 | -2.33 | 0.021 |
| *Enterobacteriacea* | 6.34E7±4.59E7 | 1.20E8±3.90E8 | 18.60 | 0.000 | -1.90 | 0.060 |
| *Prevotella spp.* | 9.10E7±1.34E8 | 4.25E8±8.44E8 | 17.14 | 0.000 | -2.47 | 0.015 |
| *Lactobacillus* spp. | 3.04E7±1.91E7 | 1.82E7±4.09E7 | 1.01 | 0.317 | 1.70 | 0.092 |
| *Clostridium* spp. | 8.68E8±1.86E9 | 1.27E9±2.30E9 | 0.27 | 0.871 | -0.86 | 0.391 |
| *Bifidobacterium* spp. | 8.73E8±1.56E9 | 4.16E8±2.97E8 | 7.009 | 0.010 | 1.81 | 0.074 |
| *F. prausnitzii* | 1.83E9±1.17E9 | 1.06E9±6.01E8 | 0.580 | 0.449 | 2.54 | 0.013 |
| *Fusobacterium* spp. | 9.12E6±1.30E7 | 3.13E7±9.67E7 | 4.15 | 0.045 | -1.44 | 0.154 |
| *A. muciniphila* | 7.04E8±8.84E8 | 4.08E8±5.82E8 | 0.603 | 0.440 | 1.76 | 0.081 |
| *Veillonella* | 9.48E6±1.99E7 | 1.00E7±1.21E7 | 1.45 | 0.231 | -0.158 | 0.875 |

**Figure S1.** Heat map showing the distribution and abundance of members of the studied intestinal bacteria (in rows) within NSCLC patients with COVID-19 samples (in columns).

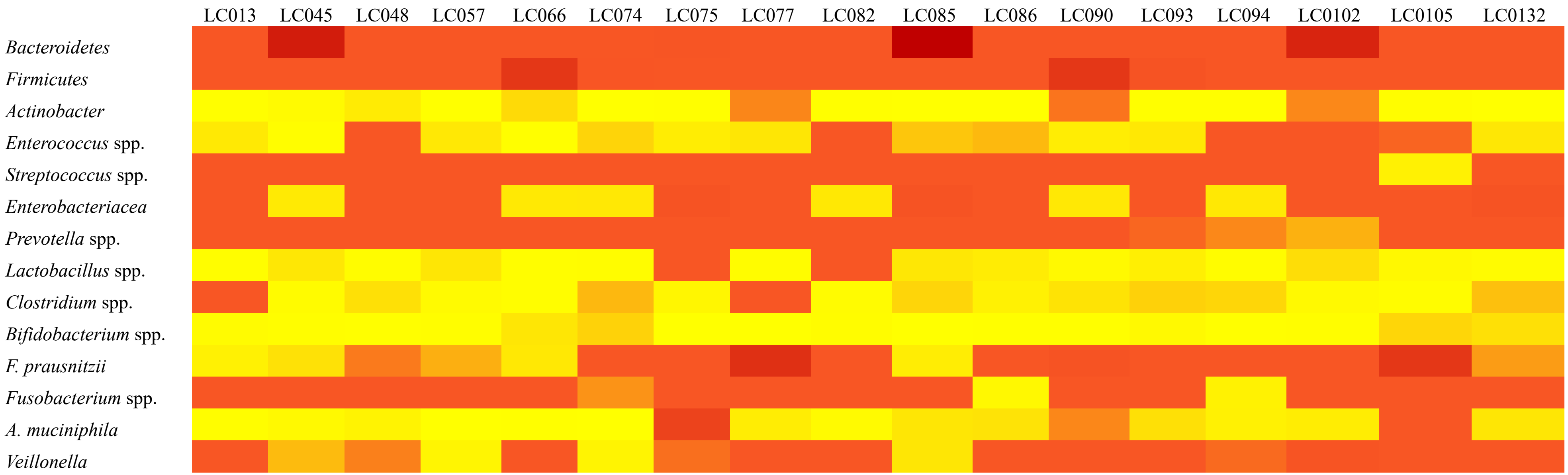

**Figure S2.** Heat map showing the distribution and abundance of members of the studied intestinal bacteria (in rows) within NSCLC patients without COVID-19 samples (in columns).

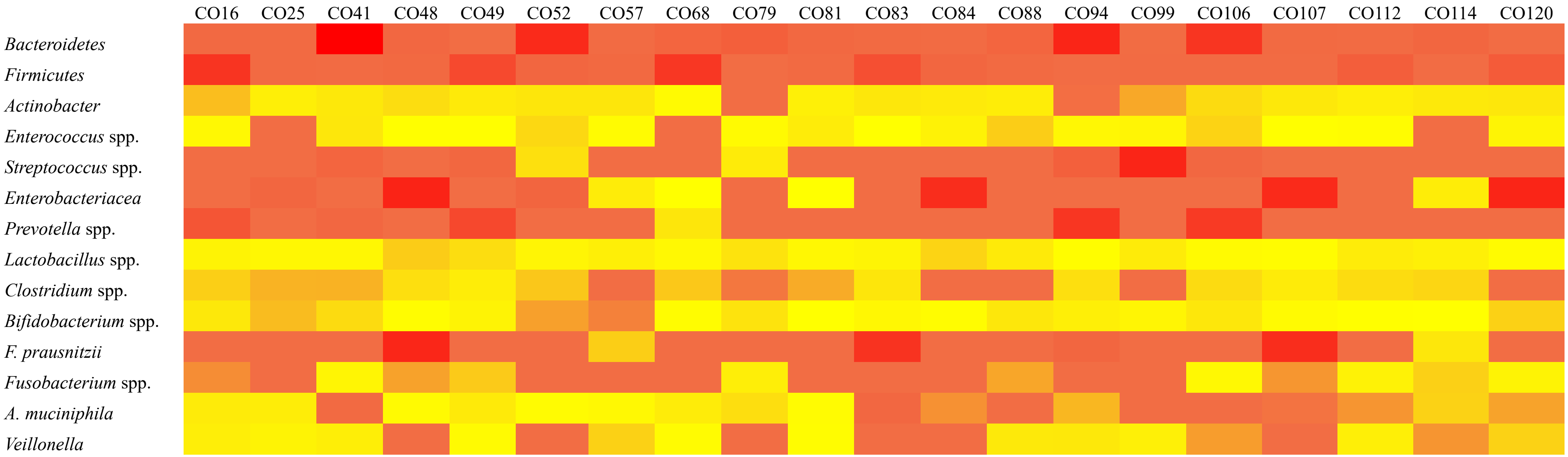

Supplement: Supplementary file 1 — contains Tables S1-S7 and Figures S1 and S2. [file aim-27-62-s001.pdf]
